# Effect of circadian clock disruption on type 2 diabetes

**DOI:** 10.3389/fphys.2024.1435848

**Published:** 2024-08-06

**Authors:** Hong Thuan Tran, Takeru Kondo, Amal Ashry, Yunyu Fu, Hiroko Okawa, Chenphop Sawangmake, Hiroshi Egusa

**Affiliations:** ^1^ Division of Molecular and Regenerative Prosthodontics, Tohoku University Graduate School of Dentistry, Sendai, Japan; ^2^ Stem Cell Institute, University of Science, Viet Nam National University Ho Chi Minh City, Ho Chi Minh, Vietnam; ^3^ Veterinary Clinical Stem Cell and Bioengineering Research Unit, Faculty of Veterinary Science, Chulalongkorn University, Bangkok, Thailand; ^4^ Veterinary Stem Cell and Bioengineering Innovation Center, Faculty of Veterinary Science, Chulalongkorn University, Bangkok, Thailand; ^5^ Department of Pharmacology, Faculty of Veterinary Science, Chulalongkorn University, Bangkok, Thailand

**Keywords:** type 2 diabetes, circadian clock, insulin secretion, insulin resistance, glucose metabolism, HPA axis

## Abstract

**Introduction:**

Type 2 diabetes (T2D) is the predominant form of diabetes mellitus and is among the leading causes of death with an increasing prevalence worldwide. However, the pathological mechanism underlying T2D remains complex and unclear. An increasing number of studies have suggested an association between circadian clock disruption and high T2D prevalence.

**Method:**

This review explores the physiological and genetic evidence underlying T2D symptoms associated with circadian clock disturbances, including insulin secretion and glucose metabolism.

**Results and Discussion:**

Notably, circadian clock disruption reduces insulin secretion and insulin sensitivity and negatively affects glucose homeostasis. The circadian clock regulates the hypothalamic–pituitary–adrenal axis, an important factor that regulates glucose metabolism and influences T2D progression. Therefore, circadian clock regulation is an attractive, novel therapeutic approach for T2D, and various circadian clock stabilizers play therapeutic roles in T2D. Lastly, this review suggests novel therapeutic and preventive approaches using circadian clock regulators for T2D.

## 1 Introduction

Diabetes mellitus is a chronic disease characterized by hyperglycemia and is classified into two major types: types 1 and 2. Type 2 diabetes (T2D) represents approximately 95% of all diagnosed diabetes cases, and its prevalence is increasing worldwide (Ong et al., 2023). The characteristic symptoms of T2D are thirst, frequent infections, and weight loss. In addition, T2D can lead to poor peripheral circulation, cardiovascular disease, kidney failure, and even death if left untreated (Chatterjee et al., 2017). However, a considerable number of cases remain undiagnosed due to the slow progression of hyperglycemia to T2D (Zheng et al., 2018). Furthermore, the pathological mechanisms underlying T2D remain complex and unclear.

Diverse environmental factors, including circadian rhythms, drive T2D pathogenesis and progression. An increasing number of studies suggest an association between circadian rhythm disruptions, such as rotating work shifts and jet lag, and a high prevalence of T2D (Pan et al., 2011; Onaolapo and Onaolapo, 2018; Gao et al., 2020). The circadian rhythm is fundamental in all living organisms, synchronizing their physiological functions with the daily environmental cycle (Reppert and Weaver, 2002). The central pacemaker of circadian rhythms is the hypothalamic suprachiasmatic nucleus (SCN) in the central nervous system. The SCN is primarily controlled by light via the retinohypothalamic tract and transmits rhythmic signals to peripheral organs (Ono et al., 2020; Jones et al., 2021). The circadian clock regulates the circadian rhythms of peripheral organs, which in turn affects metabolism, glucose intake, and insulin secretion (Stenvers et al., 2019; Parameswaran and Ray, 2022). However, the molecular mechanisms underlying the correlation between the circadian clock and T2D remain poorly understood.

The hypothalamic–pituitary–adrenal (HPA) axis is responsible for maintaining homeostasis by controlling blood glucose levels via glucocorticoid secretion (Lightman and Conway-Campbell, 2010). HPA axis dysfunction can lead to dysregulation of glucocorticoid levels, resulting in T2D progression (Li and Cummins, 2022). The HPA axis is regulated by the circadian clock; therefore, its hormone secretion exhibits circadian rhythmicity and can be modulated by day/light rhythmicity (Lightman and Conway-Campbell, 2010; Kalsbeek et al., 2012). In addition, the circadian clock controls glucocorticoid levels via the HPA axis (den Boon and Sarabdjitsingh, 2017). Thus, the circadian clock appears to be associated with T2D progression through the HPA axis.

This review summarizes the molecular mechanisms by which the circadian clock regulates blood glucose levels. Moreover, we discuss the relationship between the HPA axis and circadian clock and suggest a potential role of the circadian clock in T2D pathogenesis and progression through the HPA axis. This provides a basis for novel approaches based on regulation of the circadian clock for the prevention and treatment of T2D.

## 2 Pathophysiology of T2D

Under normal physiological conditions, increased plasma glucose levels promote insulin production and secretion from pancreatic β-cells, which stimulates blood glucose uptake into peripheral tissues and inhibits hepatic gluconeogenesis. Insulin binds to receptors on various tissues, including muscles, liver, and adipose tissue, and enhances glucose uptake into these tissues to maintain blood glucose homeostasis (Norton et al., 2022). Following uptake, glucose is used to generate energy or is stored as glycogen in the muscle and liver or as triglycerides in adipose tissue, acting as a reservoir for low blood glucose conditions (James et al., 2021).

T2D is caused by two pathophysiological conditions: pancreatic β-cell dysfunction and insulin resistance in insulin-targeting tissues. Continuous hyperglycemia promotes insulin resistance in peripheral tissues, leading to decreased sensitivity of cells to insulin. In the prediabetic stage, pancreatic β-cells increase their insulin secretion to offset insulin resistance and regulate blood glucose levels. However, chronic increased insulin secretion leads to pancreatic β-cell dysfunction and insulin deficiency, which eventually leading to the redevelopment of hyperglycemia, and ultimately T2D (DeFronzo, 2004; Muoio and Newgard, 2008).

## 3 Disruption of circadian rhythm induces T2D

### 3.1 Circadian clock regulates circadian rhythm

**FIGURE 1:**
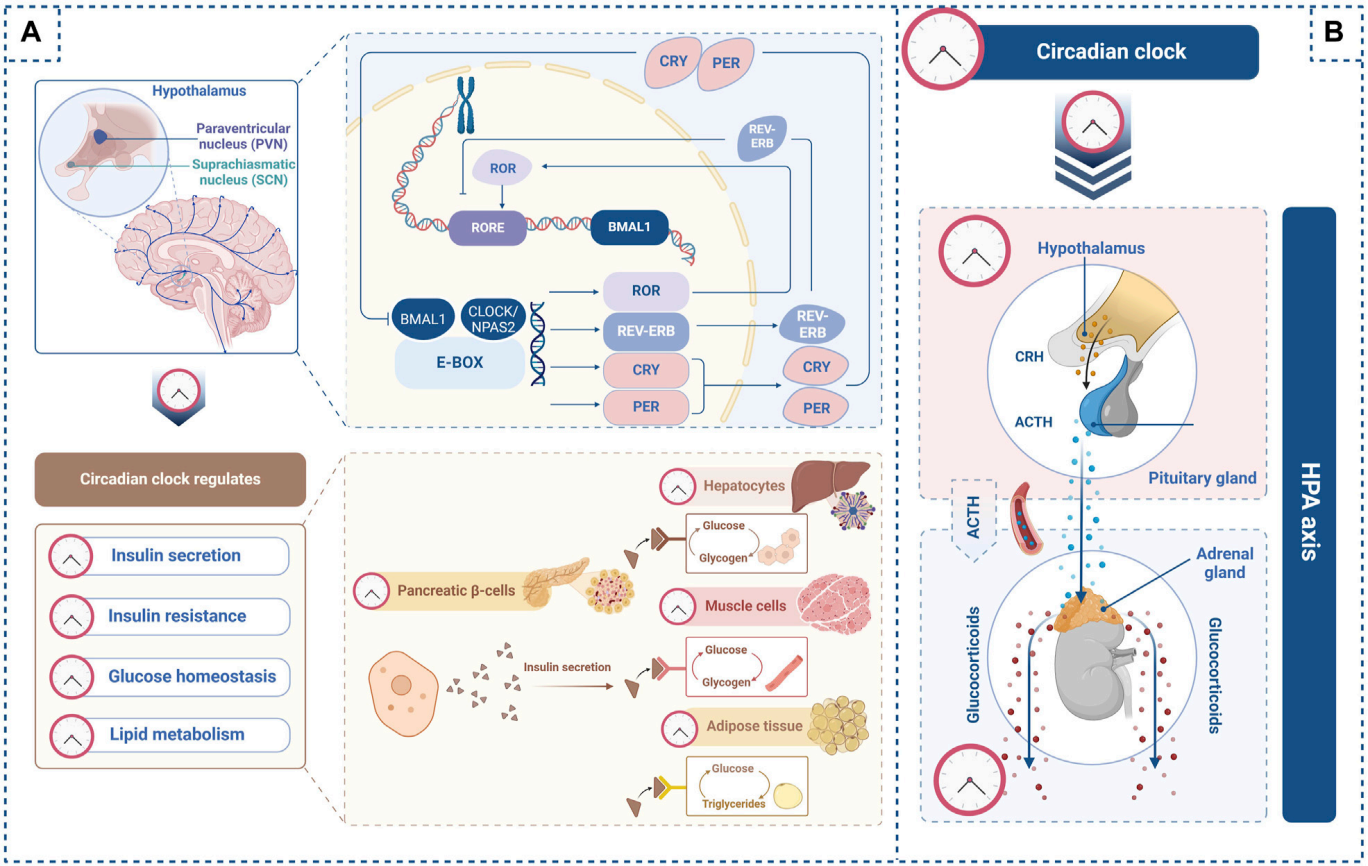
Circadian clock regulates glucose homeostasis through the hypothalamic–pituitary–adrenal (HPA) axis. **(A)** In the transcription-translation feedback loop, BMAL1 and CLOCK bind to E-box to activate *PER*, *CRY*, *ROR,* and *REV-ERB*. Npas2, a potential ortholog of CLOCK, can form a heterodimer with BMAL1. PER and CRY complexes suppress the transcriptional activation of the BMAL1 and CLOCK complex. ROR binds to the *BMAL1* promoter to enhance its transcription, whereas REV-ERB inhibits *BMAL1* gene transcription. The circadian clock regulates insulin secretion from pancreatic β-cells as well as insulin sensitivity in the liver, muscle, and adipose tissue. Furthermore, the circadian clock regulates glycogen synthesis in the liver and muscle and lipid metabolism in adipose tissues. **(B)** The circadian clock regulates the secretion of corticotropin-releasing hormone (CRH) from the paraventricular nucleus (PVN) of the hypothalamus and the secretion of adrenocorticotropic hormone (ACTH) from the pituitary gland within the HPA axis. The circadian clock also regulates glucocorticoid secretion from the adrenal glands along the HPA axis. These images were created using BioRender.com.

The circadian clock orchestrates the 24-h cycle through the transcription-translation feedback loop (TTFL) (Zhuang et al., 2023) (Figure 1A). In the TTFL, circadian clock molecules brain and muscle Arnt-like protein 1 (BMAL1) and circadian locomotor output cycle kaput (CLOCK) provide positive feedback. The BMAL1 and CLOCK heterodimer binds to a *cis*-regulatory enhancer sequence known as E-box to induce the transcription of *period* (*PER*) and *cryptochrome* (*CRY*). Complexes formed by PER and CRY translocate into the nucleus to suppress the transcriptional activation of *BMAL1* and *CLOCK*. When PER and CRY protein levels decrease, the BMAL1 and CLOCK complexes rebind to E-box and reactivate circadian clock gene transcription, initiating a new cycle. This feedback loop serves as a core regulator of the 24-h cycle (Ono, 2022).

Neuronal PAS domain protein-2 (Npas2), another circadian clock molecule, is thought to be an ortholog of CLOCK and can form a heterodimer with BMAL1 (Okawa et al., 2020). The BMAL1 and Npas2 complex may work in a compensatory manner with the BMAL1 and CLOCK complex to maintain the rhythmic cycle (Fagiani et al., 2022). Recently, Npas2 has received attention in the medical field owing to its various roles in disease progression (Morinaga et al., 2019; Yang et al., 2019).

Retinoid-related orphan receptor (ROR) and reverse orientation c-erb (REV-ERB) are other circadian rhythm regulators. ROR binds to the orphan receptor response element (RORE) in the *BMAL1* promoter to enhance its transcription, whereas REV-ERB inhibits *BMAL1* transcription (Patke et al., 2020). These subloops contribute to the 24-h rhythm by regulating molecular oscillations.

### 3.2 Circadian clock dysfunction induces T2D

Disruption of the circadian rhythm can negatively affect glucose metabolism, resulting in the promotion of diabetogenic states. Shift workers exposed to circadian rhythm misalignment show reduced insulin sensitivity and higher expression of inflammatory markers (Leproult et al., 2014). Furthermore, shift workers with circadian rhythm misalignment exhibited reduced glucose tolerance (Morris et al., 2015). Moreover, both populations with short and long sleep periods are at an increased risk of T2D progression compared with those with an optimal sleep period of 7–8 h per night (Lu et al., 2021).

The molecular mechanism of these phenomena might be explained by circadian clock function. *Bmal1* or *Clock* mutant mice exhibit T2D symptoms, including hyperglycemia and lower insulin levels. In addition, *Clock* mutant mice exhibit decreased expression of genes involved in insulin signaling, glucose sensing, and islet development (Marcheva et al., 2010). Human studies support the association between the circadian clock-associated gene variants and T2D incidence. Genome-wide association studies have demonstrated a significant link between *BMAL2* gene variants and T2D population in Japan (Yamaguchi et al., 2015). In addition, specific *BMAL1* haplotypes are reportedly strongly associated with an elevated T2D risk in the British population (Woon et al., 2007). Moreover, the *CLOCK* variant increases T2D incidence (Corella et al., 2016). Thus, circadian clock dysfunction contributes to T2D progression and is a key factor underlying the biological mechanisms involved in T2D progression.

## 4 Effects of the circadian clock on insulin secretion and resistance

### 4.1 Circadian clock regulates insulin secretion

Circadian rhythms regulate glucose metabolism, and a 24-h cycle can be detected in blood glucose and insulin levels. The insulin secretion rate peaks at noon and 6:00 p.m. and dips between midnight and 6:00 a.m. (Boden et al., 1996). The circadian clock has been demonstrated to regulate insulin secretion in pancreatic islets (Figure 1A). Rat pancreatic cells show circadian oscillations of circadian clock genes, such as *Bmal1*, *Clock*, *Per1*, *Per2*, and *CRY1,* and the output gene *Rev-erb* (Mühlbauer et al., 2004). *Clock* knockout mice show decreased proliferation and insulin secretion in pancreatic islets and hyperglycemia (Marcheva et al., 2010). In addition, in mice, pancreas-specific mutations in *Bmal1* impair normal insulin secretion (Marcheva et al., 2010; Rakshit et al., 2016), while Cry1/2 knockout results in unregulated insulin secretion (Barclay et al., 2013).

### 4.2 Circadian clock regulates insulin resistance

Insulin resistance is regulated by the circadian clock (Figure 1A), and several studies have investigated this association. Inappropriate sleep duration, late chronotype, social jetlag, and shift work are associated with the progression of insulin resistance (Stenvers et al., 2019). Individuals with the *CRY1* variant exhibit increased insulin resistance and diabetes risk compared with those without the *CRY1* variant (Dashti et al., 2014). *CLOCK* gene polymorphisms have also been closely associated with insulin resistance (Garcia-Rios et al., 2014). Additionally, *Bmal1* knockout mice display higher rates of insulin resistance compared to wild-type mice, and transgenic *Bmal1* overexpression in knockout mice ameliorates the elevated insulin resistance (Shi et al., 2013). Moreover, relative to wild-type mice, *Clock* or *Bmal1* knockdown mice exhibit insulin resistance in muscle cells (Liu et al., 2016).

## 5 Effects of circadian clock on glucose metabolism

### 5.1 Circadian clock regulates glycogen synthesis and lipid metabolism

Glycogen synthesis in the muscle and liver displays circadian rhythms and is regulated by the circadian clock of glucose homeostasis (Reinke and Asher, 2019). The circadian clock regulates glycogen synthesis by regulating the expression of glycogen synthesis-related genes, such as *glycogen synthase 2* (*GYS2*) and *phosphoenolpyruvate carboxykinase* (*PEPCK*). CLOCK can bind to the E-box located in the intron of *Gys2*, inducing *Gys2* expression (Doi et al., 2010). On the other hand, *Cry1* and *Cry2* suppress *Pepck* expression to maintain the rhythmic cycle of glycogen storage and utilization (Zhang et al., 2010; Lamia et al., 2011).

Long-term blood glucose insufficiency prompts the enzymatic hydrolysis of triglycerides stored in white adipose tissues to produce free fatty acids and glycerol, which are released into the blood as an energy source (Unger et al., 2010). However, the redundant deposition of triglycerides by continuously increasing glucose levels leads to obesity and ultimately the development of T2D. Adipose tissues show rhythmic expression of circadian clock genes that control lipid metabolism (Ando et al., 2005). In addition, disruption of the circadian clock induces uncontrolled lipid metabolism. *Clock* mutant mice exhibit lower and non-rhythmic blood free fatty acid and glycerol levels with a decreased rate of lipolysis compared with that in wild-type mice (Shostak et al., 2013). Furthermore, adipose tissue-specific *Bmal1 deficient* mice exhibit impaired triglyceride hydrolysis compared with that in wild-type mice (Paschos et al., 2012). *Rev-erb* knockout mice display higher circulating triglyceride levels and lower free fatty acid levels than wild-type mice, suggesting that *Rev-erb* deficiency results in dysregulation of lipid metabolism (Cho et al., 2012). Hence, circadian clock disruption impairs glycogen synthesis and lipid metabolism (Figure 1A), increasing the risk of T2D.

### 5.2 Insulin regulates glucose metabolism through the circadian clock

The relationship between insulin and the circadian clock is bidirectional. Insulin signaling affects the circadian clock and various downstream functions in peripheral tissues. For instance, insulin administration induces a circadian rhythm phase shift in rat liver (Yamajuku et al., 2012). Individuals with insulin resistance exhibit disrupted rhythmic patterns in the expression of muscle circadian clock-associated genes, suggesting that insulin signaling plays an important role in the circadian clock of muscles (Wefers et al., 2020). Insulin directly regulates the expression of circadian clock-associated genes in both human and mouse adipose tissues (Tuvia et al., 2021). Moreover, insulin signaling induces *Cry1* expression and regulates glucose and lipid metabolism (Jang et al., 2016). Chronic high serum insulin concentrations induced by a high-fat diet were reported to disrupt circadian rhythms, including *Bmal1* and *Clock* expression, and inhibit the expression of lipogenesis-related genes in the mouse liver when compared with mice fed a normal diet (Honma et al., 2016). Thus, insulin signaling regulates glucose metabolism by regulating the expression of circadian clock molecules.

## 6 Relationship between HPA axis and T2D

Upon activation of the HPA axis, the paraventricular nucleus (PVN) in the hypothalamus releases corticotropin-releasing hormone (CRH), which stimulates the pituitary gland to secrete adrenocorticotropin (ACTH). ACTH then travels through the systemic circulation to the adrenal cortex where it induces glucocorticoid secretion (Herman et al., 2016; Lightman et al., 2020).

Glucocorticoids are essential for living organisms under stress conditions (Sapolsky et al., 2000). However, excessive glucocorticoid levels can lead to the development of T2D through induction of hyperglycemia, insulin resistance, and dyslipidemia (van Raalte and Diamant, 2014). Glucocorticoids directly increase hepatic insulin resistance and gluconeogenesis-related enzymes in the liver, leading to hyperglycemia (Vegiopoulos and Herzig, 2007). In the adipose tissue, glucocorticoids upregulate lipid uptake, triglyceride synthesis, and lipolysis, contributing to the local free fatty acid pool (Wang et al., 2012). In addition, glucocorticoids can decrease insulin sensitivity and glucose uptake in the skeletal muscles (Ruzzin et al., 2005). Furthermore, long-term glucocorticoid exposure leads to pancreatic β-cell apoptosis and disrupts insulin secretion (Li and Cummins, 2022).

Additionally, disruption of the HPA axis results in dysregulation of glucocorticoid production, contributing to T2D progression. HPA axis dysfunction can lead to increased daily glucocorticoid levels, hyperglycemia, and insulin resistance (Gan et al., 2023). The metabolic stress in T2D can further disrupt the HPA axis, exacerbating hyperglycemia through unregulated glucocorticoid levels (Joseph and Golden, 2017).

## 7 Effect of the circadian clock on the HPA axis

HPA axis-dependent glucocorticoid release is rhythmic given that it is regulated by the light-activated master circadian clock, which is the central pacemaker in the SCN (Ishida et al., 2005). Glucocorticoid levels increase in the morning and decrease after 2–3 h of sleep (Shimba and Ikuta, 2020). The master circadian clock is thought to regulate CRH release from the PVN in the hypothalamus and ACTH secretion from the pituitary gland within the HPA axis (Kalsbeek et al., 2012). In addition, the master circadian clock controls glucocorticoid secretion from the adrenal gland in the HPA axis by regulating adrenal cortex sensitivity to ACTH (Oster et al., 2006).

Various studies have shown that the circadian clock regulates the HPA axis and glucocorticoid secretion from adrenal glands. BMAL1 is reportedly expressed in CRH neurons in the PVN (Hung et al., 2021). Furthermore, the expression of circadian clock molecules, including *Bmal1*, *CRY1*, *Per1*, and *Rev-erb,* has been observed in the adrenal glands (Dumbell et al., 2016). *Per1* mutant mice exhibit elevated glucocorticoid levels without a circadian rhythm, while *Per2* mutant mice display higher circulating glucocorticoid levels than wild-type mice (Dallmann et al., 2006).

The loss of *Bmal1* in CRH neurons causes arrhythmic activity and reduces glucocorticoid secretion (Jones et al., 2021). Additionally, adrenal gland-specific *Per2* and *Cry1* double-knockout mice exhibit impaired glucocorticoid production (Oster et al., 2006). These results suggest that the peripheral circadian clock in the HPA axis regulates glucocorticoid secretion.

Taken together, the light input-dependent master circadian clock regulates glucocorticoid secretion through activation of the HPA axis, whereas the peripheral circadian clock controls the rhythmic release of glucocorticoids from the adrenal glands (Figure 1B).

## 8 Circadian clock regulation as a therapeutic target for T2D

The circadian clock is closely associated with T2D progression. We understand that too much data comes from animal experiments at present, and the timing and composition of meals still need to be considered in humans. However, the reported results prompted interesting pharmacological investigations. In recent years, regulation of the circadian clock to control blood glucose levels has gained attention as a novel therapeutic approach for T2D (Hudec et al., 2020) (Table 1). For example, cryptochrome stabilizers would be therapeutic reagents for T2D. TW68 suppresses the gene expression of *Pepck1* and *glucose-6-phosphatase*, which play important roles in glucogenesis, by stabilizing CRY1/2, resulting in lower blood glucose levels in high-fat diet-fed mice (Surme et al., 2023). On the other hand, KL001 prevents CRY1 and CRY2 degradation, extending the circadian period, and KL001-mediated CRY stabilization suppresses hepatocyte glucogenesis (Hirota et al., 2012). Metformin, a biguanide anti-hyperglycemic agent, activates adenosine monophosphate-activated protein kinase, a cellular metabolic regulator, by upregulating *Bmal1* expression in T2D mice (Alex et al., 2020). Nobiletin, a phytochemical compound, significantly suppresses blood glucose levels and improves insulin sensitivity by upregulating the expression of various circadian clock-associated genes, including *Bmal1*, *Per2*, *Cry1*, *Cry2*, *Ror*, and *Rev-erba* (He et al., 2016). Furthermore, PF-5006739, a casein kinase 1δ and ε inhibitor, normalizes the dysregulation of various circadian clock-associated genes, including *Bmal1* and *Rev-erba,* improving glucose tolerance (Cunningham et al., 2016). Similarly, thiazolidinedione resolves the disruption of the expression of circadian clock-associated genes(*Bmal1*, *Per2*, *Cry1*), improving insulin resistance (Yang et al., 2013). Circadian clock regulators, such as antidepressants, antipsychotics, and mood stabilizers, which can regulate the expression of *Bmal1*, *Clock*, and *Per1*, decrease the incidence of HPA axis dysregulation in patients with T2D, improving their blood glucose and glycosylated hemoglobin levels (Min et al., 2023).

**TABLE 1:** Summary of studies investigating circadian clock stabilizers for type 2 diabetes.

Circadian clock stabilizer	Target circadian clock molecule	Key conclusions	References
TW68	Cry1 and Cry2	TW68 reduced blood glucose levels by enhancing CRY stabilization	Surme et al. (2023)
KL001	Cry1 and Cry2	KL001 prevented CRY degradation and inhibited glucogenesis	Hirota et al. (2012)
Metformin	Bmal1	Metformin activated AMPK, a cellular metabolic regulator, by upregulating *Bmal1* expression	Alex et al. (2020)
Nobiletin	Bmal1, Per2, Cry1, Cry2, and Rev-erba	Nobiletin suppressed blood glucose levels and improved insulin sensitivity by upregulating circadian clock-associated genes, such as *Bmal1*, *Per2*, *Cry1*, *Cry2*, and *Rev-erba*	He et al. (2016)
PF-5006739	Bmal1 and Rev-erba	PF-5006739 normalized dysregulation of *Bmal1* and *Rev-erba* and improved glucose tolerance	Cunningham et al. (2016)
Thiazolidinedione	Bmal1, Per2, and Cry1	Thiazolidinedione resolved disrupted *Bmal1*, *Per2*, and *Cry1* expression and improved insulin resistance	Yang et al. (2013)

High-throughput screening (HTS) is a promising tool for identifying novel circadian clock stabilizers for T2D treatment. Although HTS was initially used to identify new pharmacological agents (Kennedy et al., 2008), it can uncover new aspects of well-characterized chemical compounds (Entzeroth et al., 2009). Using HTS, a compound that can regulate *Npas2* expression has been identified; this compound has been demonstrated to be useful for skin wound treatment by regulating the circadian clock (Clements et al., 2022; Shibuya et al., 2022). Therefore, HTS might help identify novel compounds that may regulate target circadian molecules for T2D treatment.

## 9 Conclusion

In this review, we discuss the potential role of the circadian clock in T2D progression. Circadian clock disruption decreases insulin secretion, increases insulin resistance, and disrupts glucose homeostasis, contributing to T2D progression. Additionally, this review suggests that the circadian clock regulates the HPA axis, which is vital for glucose homeostasis and T2D progression. While current evidence suggests a link between circadian rhythm disruption, the HPA axis, and T2D, further research is necessary to fully understand the mechanisms underlying this complex link in T2D pathogenesis and progression. The use of circadian clock stabilizers is a therapeutic approach for T2D, and HTS is a useful tool for identifying novel chemical compounds for regulation of the circadian clock.
